# Does assessment drive learning or should learning for professional life drive assessment?

**DOI:** 10.3205/zma001582

**Published:** 2022-11-15

**Authors:** Katrin Schüttpelz-Brauns

**Affiliations:** 1 Universitätsmedizin Mannheim (UMM), Medizinische Fakultät Mannheim der Universität Heidelberg, Geschäftsbereich Studium und Lehrentwicklung, Abteilung Medizinische Ausbildungsforschung, Mannheim, Germany

## Editorial

During class my students ask me again and again if the topics we are discussing are relevant for the exam. These questions mainly arise in connection with such exciting topics like the explanatory power of correlation coefficients or the max-con-min principle. My response that “you need this for your life” does not increase the level of attention. In these cases it appears that the assessments are more important to the students than the content itself. And it is not just for my students that exams are an important driver of learning. In the frequently held discussions about the relationship between learning and assessing the position that is increasingly being taken is: What's not tested, won't be learned.

Indeed, it can be empirically demonstrated that assessments influence learning. A direct influence is present, for example, in test-enhanced learning, through which demonstrably more knowledge is retained, or with feedback on a (learning process-focused) assessment, through which learning behavior is adapted based on the feedback. Assessments do have effects that drive learning. This means that because students are preparing for an assessment, they select learning objectives based on the tested material and adapt their learning strategies to the assessment formats, e.g. [1]. Assessments are often associated with anxiety and pressure, e.g. [2]. As a result, assessments are not only a means to an end, but also connect an emotional cost to learning.

This leads to a consideration of learning from a motivational perspective. Two theories present themselves for this: the self-determination theory of Ryan & Deci [3] and the expectancy-value theory according to Wigfield & Eccles [4]. The self-determination theory differentiates between three forms of motivation: amotivation where there is no motivation, extrinsic motivation where the drive comes from outside the learner, and intrinsic motivation where the task itself is the drive to learn. The theory goes on to divide extrinsic motivation into four subtypes depending on the locus of the drive. Listed in order from external to internal, these are external regulation, introjected regulation, regulation through identification, and integrated regulation. When applied to learning, no learning takes place in the case of amotivation. With external regulation, students learn because they perceive pressure from third parties, and with introjected regulation because they have a bad conscience or wish to prove to others that they can do it. In the case of regulation through identification, learning is viewed as part of a freely chosen course of study. With integrated regulation the content is learned because they are preparing for (professional) life. If students are learning to get good grades or not to fail, they are learning, with a high probability, exactly what the assessment demands, in terms of both content and depth as dictated by the assessment format. Indeed, it is shown that extrinsic motivation is associated more with superficial learning strategies, such as bulimic learning, and intrinsic motivation more with elaborate learning strategies that aim for long-term retention [5]. According to the self-determination theory, more extrinsically motivated students learn for the assessments and more intrinsically motivated students learn for (professional) life or because they are interested in and enjoy grappling with the content. However, this claim cannot be made across the board. There are still other mechanisms that work to switch the focus from learning to assessment. The empirically valid expectancy-value theory postulates that achievement can be explained by motivation [4]. This results from the connection between a student's expectancy of success and the value they see in a task (e.g., an assessment). The value of the assessment is comprised of its intrinsic value, e.g., an exciting challenge, its importance/relevance, or the cost, which can also be of an emotional nature. That this cost often moves to the foreground is also shown in studies, e.g. [2]. If the value of the assessment and the emotional cost are very high, then the focus probably moves away from learning for (professional) life and more toward learning for the assessment. Theoretically, in a curriculum with constructive alignment the teaching should prepare for the assessments and this preparation should therefore primarily be a repetition of the curricular content. Indeed, I hear again and again in conversations with students that they attend classes, actively participate, but they study for the assessments independently of all that (e.g., using old tests or specific services like AMBOSS). At such a point in an education, only what is tested is learned due to the high value of the assessments and the shortage of time.

Thus, it is easy to understand that the discussion about the relationship between learning and assessment is moving more and more toward the standpoint: What's not tested, won't be learned. Nevertheless, we should systematically investigate the causes in order to refocus on education and learning. Only this way can assessments fulfill their function: to exist alongside the learning process and ensure that minimum standards are met. No more, no less.

In this issue, only one paper focuses on assessment, on a tele-OSCE in oral and maxillofacial surgery [6]. One paper focuses on students by investigating the development of depression at the beginning and over the course of medical study and identifying influencing factors, including resiliency factors [7]. Several papers focus on teaching formats, such as an interprofessional education day to impart interprofessional competencies [8], a clinical elective on domestic violence [9], interventions for dealing with the pharmaceutical industry [10], simulation-based medical education [11], and training medical students at specialized out-patient practices [12]. Two papers concern overarching topics, such as the publication activity in this journal regarding digital teaching and learning [13] and the opportunities for post-graduate training in Germany [14]. One paper scrutinizes a diagnostic instrument with a description of its development and test statistical verification as a questionnaire to measure teaching competencies in medicine [15].

## Competing interests

The author declares that she has no competing interests.
